# A Retrospective Study of 23 Cases: Are Lichenoid Lesions of the Labial Mucosa Induced?

**DOI:** 10.7759/cureus.25012

**Published:** 2022-05-15

**Authors:** Jean Lehner, Scarlette Agbo-Godeau, Chloé Bertolus

**Affiliations:** 1 Oral Surgery, Université Paris-Sorbonne, Paris, FRA; 2 Département de Pathologie de la Muqueuse Buccale, Service de Chirurgie Maxillo-Faciale et Stomatologie, Pitié-Salpêtrière Hospital, Paris, FRA

**Keywords:** koebner phenomenon, lip lesions, clinical research, oral lichenoid lesions, lichen planus, oral lichen planus, buccal dermatology

## Abstract

Background

Lichen planus (LP) is a pathology that affects the skin and the mucosa. The lips are rarely involved but represent a diagnostic challenge in those cases. Oral lichenoid lesions (OLL) are defined as lesions that resemble oral lichen planus (OLP) but do not fully meet the clinical and/or histologic criteria for OLP. This study aimed to present our case series and to study the correlation between the location of the lesion and the dental factor (resin composite, amalgams, crowns, abrasive teeth, and mandibular crossbite) that could cause the lesion.

Methods

We conducted a retrospective observational study of 23 patients with LP/OLL of the lips treated in the Department of Oral Mucosal Pathology of the Department of Stomatology and Maxillofacial Surgery of the Pitié-Salpêtrière Hospital in Paris between January 2017 and February 2021. We noted the location of the lesion (upper, lower, or both lips), medical history, treatments, smoking habits, and the aspect of the teeth facing the lesion. Patients received a local corticoid treatment and were monitored via follow-up.

Results

Sixteen patients had lesions on the upper lip, two on the lower lip, and five on both lips, and most patients (n = 14, 60.1%) had a dental factor facing the lesion (e.g., abrasive teeth, resin composites, dental crowns, and mandibular crossbite). Six patients received clobetasol propionate, and 15 patients received a preparation combining betamethasone and benzocaine (Orabase, ConvaTec, Deeside, UK). Fourteen patients returned for post-treatment follow-up consultations approximately two months after treatment. Seven patients saw clinical improvement, five had partial improvement, and two had no improvement.

Conclusions

Lesions of the labial mucosa appear to be a rare condition in LP/OLL. The difference between LP and OLL can be difficult, even with histological analysis. Its pathogenesis remains unknown, although some studies found evidence of lichenoid reactions of the lips in contact with dental composite restorations. In our study, 14 of our patients had a dental factor facing the lesions. However, our study failed to show a correlation between the presence of an inducing factor and the lesion. In a future study, the potential effect of dental inducing factor removal could be studied. This topic requires further investigations, particularly regarding the inducing factor and the optimal therapeutic approach.

## Introduction

Lichen planus (LP) is a chronic inflammatory dermatosis whose precise etiology is unknown, although an autoimmune factor might play a role in its occurrence [1]. This pathology affects the skin and squamous mucosa (e.g., oral, genital, and esophageal) [2]. LP is classified as oral lichen planus (OLP) or oral lichenoid lesion (OLL) [3]. OLP has a prevalence of 0.5-2% in the general population [4,5]. Women aged 30-60 years are most affected, and children are rarely affected [6].

OLP can affect all parts of the oral cavity, but the most frequently affected sites are the tongue, floor of the mouth, the inner cheeks, and the attached gingiva [7]. Symptoms may vary from absence of pain to severe pain or burning. OLP may present in different clinical forms, even in the same patient. Clinical and histological criteria define OLP. Clinically, patients exhibit bilateral and symmetrical papular and reticular whitish lesions. Histologically, the tissue will display parakeratosis, hyperkeratosis, hyperacanthosis, and hypergranulosis, with a well-defined, band-like zone of cellular infiltration confined to the superficial part of the connective tissue. This zone mainly consists of lymphocytes and small colloid cavities (Civatte bodies) [8].

OLLs are defined as lesions that resemble OLP but do not fully meet the clinical and/or histologic criteria for OLP [1] and are described in varying clinical forms [9]. Histologically, there seems to be a controversy between OLP and OLL. Among the many studies performed, some find differences [10,11] in histopathologic criteria between OLP and OLL (such as a more diffuse subepithelial infiltrate containing eosinophils and lymphocytes cells, with deeper extension), while others find similarities [12].

OLL seems to be caused by something that irritates the mucosa, especially dental amalgams [1], and the condition improves after removing the inducing factor [13,14]. These lesions are often unilateral, in contact with the restorative material, and, in one study, the removal of the irritating factor improved approximately 97% of cases [15]. OLL can be caused by systemic drug exposure (such as nonsteroidal anti-inflammatory drugs and antimalarials) [16-18].

Labial involvement of LP is rare [19] and is sometimes the only visible manifestation of the disease. It is described essentially on the lower vermilion; isolated involvement of the upper vermilion is rarely reported [7]. Isolated involvement of the labial mucosa has not been reported. The diagnosis is difficult when the labial involvement is isolated because of the similarities with the etiology of cheilitis. The differential diagnoses for OLP in lip location are discoid lupus [20] and actinic cheilitis [21]. The clinical appearance most often observed on the lips is a small erythematous area, sometimes erosive, surrounded by keratotic streaks. The appearance might also be annular [22]. The labial localization of bullous LP is very rare [23]. We conducted this study to determine the correlation between the location of the lesion and potential dental causative factors.

## Materials and methods

Aim and objectives

The aim of this study was to report our case series of 23 patients with labial lesions of LP/OLL, and to study the correlation between lip lesion and a dental factor that could cause the lesion.

Study design

We present a retrospective observational study of patients with LP/OLL of the labial mucosa who presented to the Department of Oral Mucosal Pathology in the Department of Stomatology and Maxillofacial Surgery of the Pitié-Salpêtrière Hospital in Paris between January 2017 and February 2021.

Inclusion criteria

Patients were included in the study if they were aged 18 years or older and had a labial lesion of LP/OLL. Other lesions of oral LP, if present, were noted. The diagnosis was performed by a skilled practitioner in oral medicine.

Exclusion criteria

Patients were excluded from the study if they were younger than 18 years, had a history of oral or lip cancer, or had a history of head and neck radiosurgery.

IRB approval

The study was approved by the general register of Paris Hospital (Registre Général des Traitements de l’Assistance Publique des Hopitaux de Paris: 20210729155806).

Data collection

We reviewed patients' medical records and collected patients' data on chief complaints, demographic characteristics (e.g., age and sex), medical history, and treatments. We also noted patients' smoking habits (quantified as packs per year), allergies, time from onset of pain to consultation (in months), symptoms, associated symptoms, and duration.

The location of the lesion and the presence of other associated intraoral lesions were collected. We also noted the aspect of the teeth in relation to the lesions or any factor that could irritate the mucosa. Histopathology findings, when available, were noted.

All patients received a local corticoid treatment, i.e., clobetasol propionate or a magistral preparation combining Diprolene/Orabase (benzocaine; ConvaTec, Deeside, UK), applied twice daily for two months. We also noted clinical signs and symptoms during follow-up visits.

## Results

Twenty-three patients were included in the study (14 women and nine men). The mean age was 67 years (±9.1 years SD). Five patients were former smokers, five had type 2 diabetes, three had a history of hypothyroidism, one patient had a history of hepatitis C virus infection, and none reported any allergies.

Table 1 presents all participants' demographics, lesion information, histopathology, treatment, and outcomes. Twelve patients had pain that interfered with eating and speaking localized to the upper lip, two patients had pain localized to the lower lip, and three patients had pain in both lips. Three patients reported discomfort in the upper lip, and three reported no pain. The average duration of pain was 21.2 months at the time of consultation.

**Table 1:** Patients' chief complaints, demographics, lesions, histopathology, treatment, and outcomes data. F, female; M, male; N/A, not applicable; HCV, hepatitis C virus; LP, lichen planus.

Sex	Age at diagnosis (years)	Patient’s chief complaint	Medical history	Lip involved	Smoking (packs/ year)	Time from pain onset to consultation (months)	Histopathology	Associated lesions	Treatment received	Response to treatment
F	74	Lip pain	0	Lower lip	0	1	N/A	Tongue tip	Betamethasone/Orabase	N/A
M	80	Lip pain	Diabetes	Lower lip	0	7	N/A	Buccal mucosa	Clobetasol	Partial remission
M	57	Lip pain	0	Upper lip	0	12	N/A	Buccal mucosa	Betamethasone/Orabase	Partial remission
F	75	Lip pain	0	Upper lip	0	5	N/A	Buccal mucosa	Betamethasone/Orabase	N/A
M	54	Lip pain	0	Upper lip	0	36	LP	0	Betamethasone/Orabase	N/A
M	68	Lip pain	0	Upper and lower lip	0	5	N/A	Buccal mucosa	Betamethasone/Orabase	Significant improvement
M	48	Lip lesion	0	Upper and lower lip	10	N/A	N/A	Tongue tip	Betamethasone/Orabase	N/A
F	81	Lip pain	0	Upper lip	0	72	N/A	0	Betamethasone/Orabase	No response
M	82	Lip lesion	0	Upper and lower lip	5	4	LP	Tongue tip	Clobetasol	N/A
F	70	Lip pain	0	Upper lip	18	12	N/A	Lateral tongue margin	Betamethasone/Orabase	Partial remission
F	53	Lip pain	0	Upper lip	0	N/A	N/A	Superior gingival mucosa	Clobetasol	Significant improvement
F	57	Lip pain	Diabetes	Upper lip	0	6	N/A	0	Betamethasone/Orabase	N/A
F	54	Lip pain	0	Upper lip	0	4	LP	0	Clobetasol	No response
M	66	Lip pain	HCV	Upper lip	0	3	N/A	Buccal mucosa (right)	Clobetasol	Partial remission
M	62	Lip pain	Diabetes	Upper lip	0	2	N/A	Superior gingival mucosa	Clobetasol	Significant improvement
F	89	Lip pain	Diabetes	Upper lip	0	84	N/A	Superior gingival mucosa	Betamethasone/Orabase	N/A
F	64	Lip lesion	0	Upper lip	0	1	LP	0	N/A	N/A
F	65	Lip pain	Psoriasis	Upper and lower lip	0	12	N/A	Lateral tongue margin	Betamethasone/Orabase	Significant improvement
M	56	Lip lesion	0	Upper lip	0	6	N/A	0	Betamethasone/Orabase	Significant improvement
F	71	Lip lesion	Hypothyroidism, psoriasis	Upper lip	6	36	N/A	0	Betamethasone/Orabase	Significant improvement
F	82	Lip pain	Hypothyroidism	Upper lip	0	6	LP	Buccal mucosa	Clobetasol	N/A
F	76	Lip pain	Hypothyroidism	Upper lip	0	24	N/A	Superior gingival mucosa	Betamethasone/Orabase	Partial remission
F	61	Lip lesion	Diabetes, hypothyroidism	Upper and lower lip	0	72	N/A	Buccal mucosa	Betamethasone/Orabase	Significant improvement

Five patients reported concerns of episodic lip swelling, three reported burning sensations, and two reported discomfort when eating spicy foods. Most lesions were located only on the upper lip (n = 16, 69%), five patients had lesions on both lips (21.8%), while only two patients had lesions only on the lower lip (8.7%). The clinical form most often found was erythematous (78%) and erosive (22%).

Other localizations of OLP were minimal and asymptomatic. Five patients had OLP on the inner surface of the cheeks (21.8%), three on the tip of the tongue (13%), five on the vestibular gingiva opposing the affected lip (21.8%), two on the lateral edges of the tongue (8.7%), one on the ventral surface of the tongue (4.3%), and one on the labial commissure (4.3%).

Six patients had resin composite teeth adjacent to the lesion (26.1%), three had abrasive teeth by the lesion (13%), three had crowns (13%), five had healthy teeth (21.7%), two had amalgams (9.1%) (distant from the lesion), one had a mandibular crossbite (4.3%), and one had a fissured tooth (4.3%). For two remaining patients, this information was missing. Fourteen patients (60.1%) had a dental factor facing the lesion (abrasive teeth, resin composites, and dental crowns). Five patients had a biopsy performed at the diagnosis, which indicated LP (band-like zone of cellular infiltration, hyperkeratosis, and hyperacanthosis). Table 2 presents patients' symptoms, associated symptoms, lip involved, dental factors, and their location.

**Table 2:** Patient demographics, symptoms presentation, associated symptoms, lip involved, dental factor, and teeth number. F, female; M, male; N/A, not applicable.

Sex	Age at diagnosis (years)	Symptoms presentation	Associated symptoms	Lip involved	Dental factor opposing the teeth	Teeth number	Possible correlation between the dental factor and lip lesion	Associated lesions
F	74	Lower lip pain	0	Lower lip	Normal	N/A	N/A	Tongue tip
M	80	Lower lip pain	Burning sensation	Lower lip	Abrasive teeth	11, 21, 22	Yes	Buccal mucosa
M	57	Upper lip pain	0	Upper lip	Normal	N/A	N/A	Buccal mucosa
F	75	Upper lip pain	0	Upper lip	Dental crowns	11, 12	Yes	Buccal mucosa
M	54	Upper lip pain	Lip swelling	Upper lip	Dental plaque	11, 12, 13, 21, 22	Yes	0
M	68	Upper and lower lip pain	0	Upper and lower lip	Amalgam on tooth	27	No	Buccal mucosa
M	48	No pain	Lip swelling	Upper and lower lip	Resin composite on teeth	11, 21, 12, 22	Yes	Tongue tip
F	81	Upper lip pain	0	Upper lip	Abrasive tooth and dental plaque	11, 12	Yes	0
M	82	Discomfort in the upper lip	0	Upper and lower lip	Normal	N/A	N/A	Tongue tip
F	70	Upper lip pain	Discomfort with spicy food	Upper lip	Resin composite	11, 21	Yes	Lateral tongue margin
F	53	Upper lip pain	Burning sensation	Upper lip	Resin composite and dental plaque	11, 12, 23	Yes	Superior gingival mucosa
F	57	Upper lip pain	Lip swelling	Upper lip	Crowns	21, 22, 24, 26	Partially	0
F	54	Upper lip pain	0	Upper lip	Resin composite	12, 22	Yes	0
M	66	Upper lip pain	0	Upper lip	Amalgam	16	No	Buccal mucosa (right)
M	62	Upper lip pain	0	Upper lip	Resin composite	22	Yes	Superior gingival mucosa
F	89	Upper lip pain	0	Upper lip	Normal	N/A	N/A	Superior gingival mucosa
F	64	No pain	Lip swelling	Upper lip	Resin composite	22	Yes	0
F	65	Upper and lower lip pain	Discomfort with spicy food	Upper and lower lip	Crowns	11, 12, 21, 22	Yes	Lateral tongue margin
M	56	Discomfort in the upper lip	0	Upper lip	Abrasive tooth (enamel defect)	11	Yes	0
F	71	Discomfort in the upper lip	0	Upper lip	Normal	N/A	N/A	0
F	82	Upper lip pain	0	Upper lip	Fissured teeth and dental plaque	11	Yes	Buccal mucosa
F	76	No pain	Burning sensation	Upper lip	Fissured teeth	11	Yes	Superior gingival mucosa
F	61	Upper and lower lip pain	Lip swelling	Upper and lower lip	Mandibular crossbite	11, 12, 13, 21, 22, 23	Yes	Buccal mucosa

All patients received local corticosteroid treatment applied twice daily; six received clobetasol propionate, and 15 received a preparation combining betamethasone and benzocaine (Orabase). Fourteen patients were reviewed in a post-treatment follow-up consultation. The follow-up timing was varied, but the mean time to follow-up was two months after the initial consultation. Seven patients showed improvement, five showed partial improvement, and two had not improved.

## Discussion

**Figure 1:**
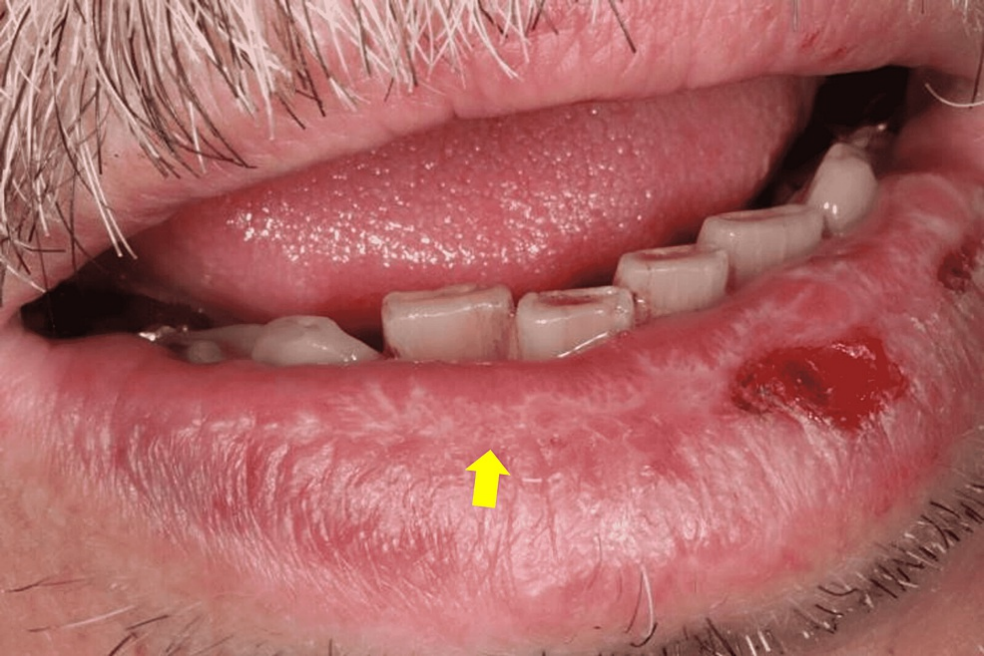
Oral lichen planus (OLP) of the lower lip. The erythematous area is surrounded by keratotic streaks.

In our study, the mean age of the patients was 67 years (±9.1 SD), which is in accordance with Nuzzolo et al.'s results [24]. Most of our patients had lesions on the mucosal side of the upper lip (n = 16, 59.6%) or on both lips (n = 5, 21.7%). Few articles report this presentation of OLP. In 2018, Katsoulas et al. [12] studied 24 patients presenting with lichenoid lesions of the upper lip. Out of 24 patients, they reported resin composites in six patients' teeth facing the lesions. In a small case series, Petruzzi et al. [25] reported that in 10 patients, most lesions were on the lower lip (n = 7) or both lips (n = 3). The predominance of vermilion lesions on the lower lip (Figure 1) might be due to exposure to ultraviolet radiation, food irritations, and saliva.

Nuzzolo et al. [24], in a retrospective clinical study in 2016, found in a case series of 13 patients an LP predilection for lower lip involvement (9:1 ratio). The major limitation of these precedent studies is the size of the sample, which was also a limitation in our study. The prevalence of LP of the lips, assessed in a study by Xue et al. [7], seems to be low (8.9% of 674 patients).

The lip location of the lesions in our study raises the question of inducing factors and the diagnosis of OLP or OLL. The distinction between LP and OLL can be difficult, even when histopathology is performed. Mravak-Stipetic et al. reported a correlation between clinical and pathological diagnosis in 52.5% of LP cases and in 42.9% of OLL cases studied [10]. The French guidelines for the management of OLP introduce a new generic definition of oral lichen, including LP and OLL [26].

The Koebner phenomenon is the appearance of skin lesions after trauma in a patient with an underlying dermatosis and has been described in patients with psoriasis, LP, and vitiligo [27,28]. These lesions are clinically and histologically identical to those of the underlying pathology.

Dental factors causing mucosal irritation might be a cause of these lesions. In the literature, studies report lichenoid lesions of the lips in contact with composite restorations [14,29]. Blomgren et al. [14] found in their study that the replacement of dental materials associated with antifungal treatment led to healing in seven out of nine patients with OLL of the lips. Dental restorative materials could also trigger an allergic reaction through a lymphocyte-mediated delayed hypersensitivity [30]. A study by Issa et al. in 2005 showed that the replacement of dental restorations could result in the improvement or resolution of OLL in most cases [30].

**Figure 2:**
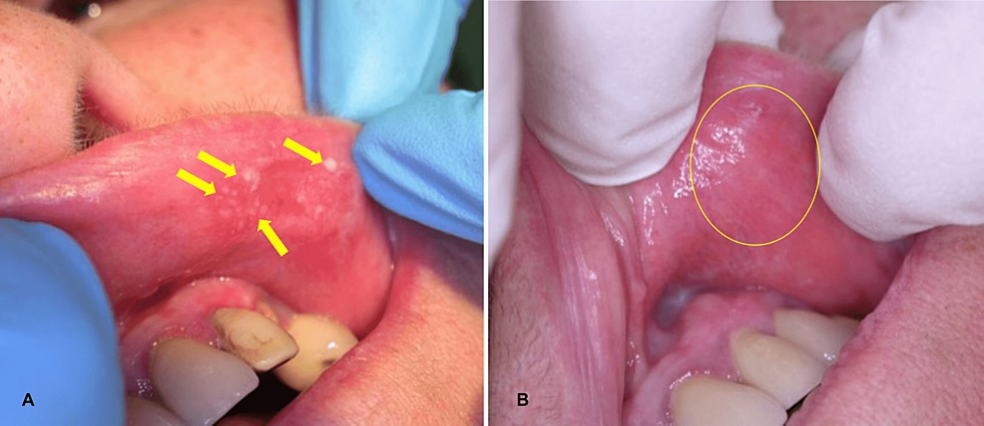
(A) Initial aspect, oral lichenoid lesion (OLL) of the upper lip. (B) Aspect after resin composite replacement. A: Old resin composite on tooth 22, OLL of the upper lip. B: Healing of the lesion.

In one patient, the replacement of an old metal-ceramic crown (tooth 23) and the removal of an old dental composite (tooth 22) resulted in the disappearance of the lesion on the upper lip (Figure 2).

**Figure 3:**
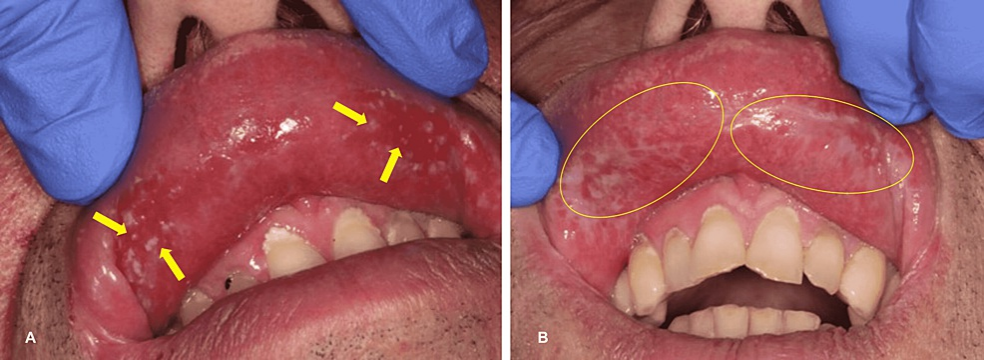
(A) Oral lichenoid lesion (OLL) of the upper lip before medical treatment. (B) OLL of the upper lip after medical treatment. A: Erythematous and erosive lesion of the upper lip. B: Improvement of the lesion after local corticosteroids.

These lesions, which are often painful and incapacitating, are improved by treatment with local corticosteroids, but no study participants were cured, and they required maintenance treatment with local corticosteroids (Figure 3).

Our study suffered from many limitations. First, only one patient benefited from dental care, which is one of the main limitations of our work. It could have been interesting to remove the dental factor and monitor the mucosal healing. Five patients had a biopsy performed, which is another major limitation of our study. We could not distinguish if lesions were oral LP or OLL. Due to the retrospective nature of our study, the follow-up of patients was incomplete, with only 14 patients presenting at the follow-up visit. Due to the nature of the study, the size of the sample, and the absence of a control group, statistical analysis for correlation between dental factors and lesions could not be performed.

Suppressing irritative factors, such as replacing old dental material opposing lesions, should be considered and should be studied in future research.

## Conclusions

Lesions of the labial mucosa appear to be a rare condition in LP/OLL. The difference between LP and OLL can be difficult, even with histological analysis. Its pathogenesis remains unknown, although some studies found evidence of lichenoid reactions of the lips in contact with dental composite restorations. In our study, 14 of our patients had a dental factor facing the lesions. However, our study failed to show a correlation between the presence of an inducing factor and the lesion. In a future study, the potential effect of dental inducing factor removal could be studied. This topic requires further investigations, particularly regarding the inducing factor and the optimal therapeutic approach.
